# Cognitive model construction and assessment of data analysis ability based on CDA

**DOI:** 10.3389/fpsyg.2022.1009142

**Published:** 2022-11-15

**Authors:** Xiaopeng Wu, Yi Zhang, Rongxiu Wu, Xiuxiu Tang, Tianshu Xu

**Affiliations:** ^1^Faculty of Education, Northeast Normal University, Changchun, China; ^2^School of Mathematics and Statistics, Qiannan Normal University for Nationalities, Duyun, China; ^3^School of Mathematical Sciences, East China Normal University, Shanghai, China; ^4^Science Education Department, Harvard-Smithsonian Center for Astrophysics, Harvard University, Cambridge, MA, United States; ^5^College of Education, Purdue University, West Lafayette, IN, United States

**Keywords:** data analysis ability, cognitive diagnostic assessment, math education, ability assessment, cognitive model

## Abstract

Ability of data analysis, as one of the essential core qualities of modern citizens, has received widespread attention from the international education community. How to evaluate students’ data analysis ability and obtain the detailed diagnosis information is one of the key issues for schools to improve education quality. With an employment of cognitive diagnostic assessment (CDA) as the basic theoretical framework, this study constructed the cognitive model of data analysis ability for 503 Grade 9 students in China. The follow-up analyses including the learning path, learning progression and corresponding personalized assessment were also provided. The result indicated that first, almost all the students had the data awareness. Furthermore, the probability of mastering the attribute *Interpretation and inference of data* was relatively low with only 60% or so. Also, the probabilities of mastering the rest of attributes were about 70% on average. It was expected that this study would provide a new cognitive diagnostic perspective on the assessment of students’ essential data analysis abilities.

## Introduction

### Importance of data analysis ability

With the arrival of big data era, substantial changes have taken place in different fields of society, such as the emergence of artificial intelligence, machine learning, precision medicine, and computational education (François and Monteiro, 2018). Data has become an essential asset in various professions for daily use, and data analysis ability correspondingly becomes a necessity for our work and life (Dani and Joan, 2004). In fields such as business, economics, and others, they increasingly rely on data analytics rather than experience or intuition only to make decisions in management (Bryant et al., 2008). Due to the popularity of data in daily life and workplace, data literacy has gradually become an ability that everyone shall have instead of an ability mastered by only few senior personnel in some specific industries (Borges-Rey, 2017; Sharma, 2017). Prado and Marzal (2013) have provided a list of standards for data literacy, which include (1) Determining the context of data production and reuse, as well as the value, category and format of data; (2) Figuring out when you need data and obtain it appropriately; (3) Properly evaluating data and data sources; (4) Using certain plans, measures, system architecture and appropriate evaluation methods to determine the appropriate methods to operate and analyze the data; (5) Visualization of data analysis results; (6) Using the analysis results to learn, make decisions, or solve certain problems. This list covers almost all required in data literacy and has become one of the basic qualities necessary for the future use. Moreover, data literacy also plays a crucial role in mathematics. Generation and solution of some certain mathematical problems are based on data analysis as well. Good data analysis ability can effectively help students find and solve problems. Therefore, it is of far-reaching significance to focus on cultivating students’ data analysis ability.

### Definition of data analysis ability

Commonly cultivated by means of statistical disciplines or statistical content, data analysis ability tightly relates with statistics and can be extracted from statistical literacy. However, statistical literacy so far has not been well defined and there is no consensus achieved on its definition among statistics educators, statisticians, and researchers globally (Kaplan and Thorpe, 2010; Schield, 2010; Ridgway et al., 2011; English, 2014). Moore and Cobb (2000) took “change” as the core element of statistical thought and believed that “change” was everywhere in the process. Wallman (1993) stated that statistical literacy was the ability to understand and critically evaluate the statistical results that permeate our daily lives, as well as the ability to understand the contributions of statistical thinking in the public, professional, personal, and private spheres. Through the literature, we can see that there exist common core elements in both the statistical literacy and data analysis ability. Pedagogically, data analysis offers an opportunity for students to explore openly. Conceptually, data analysis examines patterns, hubs, clusters, gaps, dissemination, and variation in data. Philosophically, some advocates of data analysis recommend introducing data in a non-probabilistic environment, while others suggest establishing a link between data analysis and the notion of probability. In the latter view, both data and contingency were considered in the framework of a systematic study of probability (Shaughnessy et al., 1996). In this study, we extracted the data analysis ability from the statistical literacy as the evidence for its definition. Specifically, we defined data analysis ability as the ability to perceive data from daily life, to consciously collect and organize data, to represent data according to different needs, and to rationally analyze and interpret data through operations.

### Assessment of data analysis ability

In the assessment of data analysis ability, Graham et al. (2009) built a framework representing children’s statistical thinking based on the Cognitive Diagnostic Model (CDM) and other relevant research. This framework provided the theory for the characterization of children’s statistical thinking and planning guidance for data processing. Reading (2002) established a four-level statistical thinking analysis framework, which were *Data Collection*, *Data Tabulation and Representation*, *Data Reduction*, and *Interpretation and Inference*. Using the Structure of the Observed Learning Outcome (SOLO, Biggs and Collis, 2014) classification framework, he analyzed the typical responses of students in different levels and drew corresponding conclusions. Mooney (2002) portrayed statistical thinking into four dimensions: *Describing data*, *Organizing and Generalizing data*, *Representing data*, and *Analyzing and Interpreting data*. Still based on the SOLO classification framework, four levels were described for each aspect: *Idiosyncratic*, *Transitional*, *Quantitative*, and *Analytical*. What’s more, they also pointed out the necessity of establishing learning trajectories that connect students with different levels of statistical thinking. Due to the high expectation in statistics teaching (Jacobe et al., 2014), effective assessment tools were in a great necessity to evaluate learners’ understanding of statistical concepts more precisely.

As we can see, people have sufficiently realized the significance of data analysis and put the cultivation of students’ data analysis ability in a more centered position. Although there is no clear definition of data analysis ability yet, a lot of research has been conducted on its attribute division, which lays the foundation for future research. Currently, the assessment of students’ data analysis ability has mostly constructed the students’ levels of abilities according to the traditional Classical Testing Theory (CTT). It exhibits great advantages in understanding the overall status of students’ data analysis ability, however, it cannot provide students with more fine-grained diagnostic information, which is the key to promoting students’ development (Huff and Goodman, 2007). The new Cognitive Diagnostic Assessment (CDA), a measurement tool developed in the most recent years combining both cognitive psychology and modern psychometrics, can analyze students’ knowledge or skill attributes involved in the process of answering questions from the perspective of cognitive psychology, and integrate attributes into the measurement model. The individual’s psychological cognitive process is measured to determine students’ mastery of attributes. CDA provides students with more detailed diagnostic information and supports a further in-depth study of students’ cognitive process (Leighton and Gierl, 2007). In this study, CDA was applied to construct a cognitive model of students’ data analysis ability. Through an examination of 503 Grade 9 students in China, the probability of mastering the attributes of students’ data analysis ability can be obtained. On this basis, the students’ learning path, learning progression, and personal assessment were then explored, providing a reference for the further study of students’ data analysis ability.

## Construction of cognitive model of data analysis ability

### Sorting out the attributes of data analysis ability

In the attribute classification of data analysis ability, data processing includes organization, description, presentation, and analysis of data, which strongly depends on the representation of various graphics and icons (Shaughnessy et al., 1996). Jones et al. (2000) formed a model composed four attributes of data analysis ability, namely, *Data organization*, *Data representation*, *Data analysis*, and *Data interpretation*. In this model, the continual process of data analysis was systematically presented. What’s more, they also made detailed operational definitions for each attribute and coded them accordingly. Mooney (2002) portrayed statistical thinking as four dimensions: *Data description*, *Data organization and generalization*, *Data representation*, *Data analysis*, and *interpretation*. Reading (2002) concluded a few steps in general, which included *Data collection*, *Data tabulation and presentation*, *Data summarization*, *interpretation*, and *inference* in the established statistical thinking framework. Arican and Kuzu (2020) divided data analysis literacy into four aspects: *(a) Data representation and interpretation, (b) Sample interpretation, (c) Statistical methods selection, (d) Understanding and application.* These studies provided the basis for the classification of data analysis ability attributes.

### The expert method

The construction of the cognitive model, especially the construction of the relationship between the attributes in the cognitive model, is more complex. Normally, expert method is used to analyze the cognitive process of students in a certain field, to obtain the relationship between different attributes. In this study, five experts were selected, including three middle school math teachers who were awarded the title of Guizhou Provincial or Municipal Famous Teachers, with rich teaching experience and knowledge of students. Another expert was a mathematics education researcher who focuses on the assessment of data analysis ability and can inspect the cognitive model of data analysis ability from the theoretical level. The last expert we invited was a doctoral student majoring in mathematics education, who has been engaging in the teaching of data problem-solving for a long time, and can examine the cognitive model of data analysis ability from the perspective of assessment.

Through open questionnaires, experts were required to enumerate the attributes of data analysis ability and draw the structural relationships between the attributes. Results of the expert method were presented in Table 1.

**Table 1:** Analysis of the expert survey of the cognitive model of data analysis ability.

No	Attribute	Structure	Common elements	Relationship
1	①Ask a question ②Collect data ③Description of data ④Amount of data concentration ⑤Discrete amount of data ⑥Make a decision	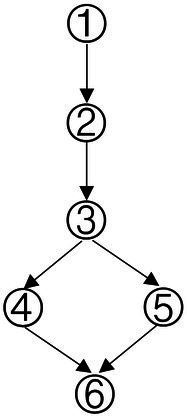	②Data collection and sorting ④The degree of data concentration ⑤The degree of dispersion of data ⑥Inference and interpretation of data	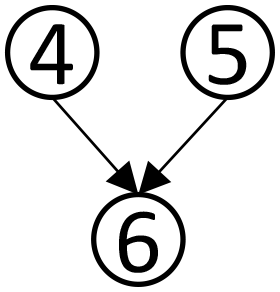
2	①Data awareness ②Data collection ③Data representation ④Understand the randomness of data ⑤Analysis means or strategy choice ⑥Interpretation of results ⑦Result presentation	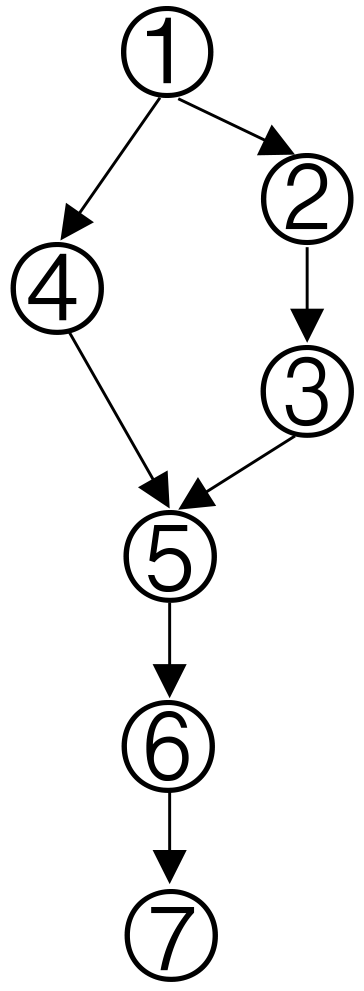	①Awareness of data ②Data collection and arrangement ③Display of data ⑥Reasoning and interpretation of data	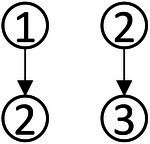
3	①Collect and organize data ②Descriptive data ③Analyze the data ④Degree of data concentration ⑤Degree of dispersion of data ⑥Experience the randomness of data	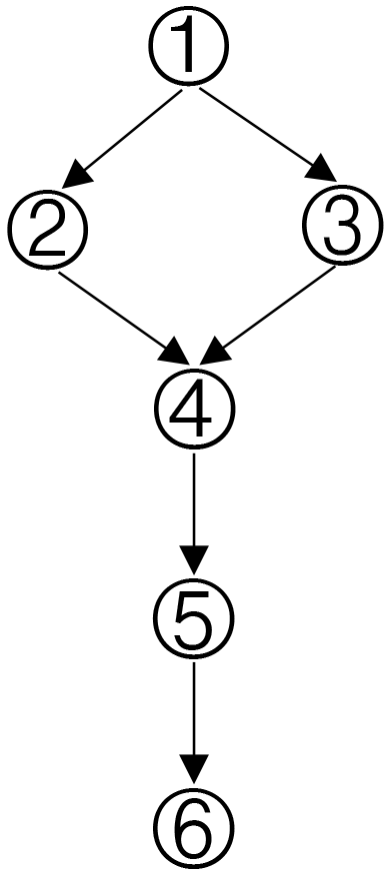	②Data collection and arrangement ④The degree of data concentration ⑤The degree of dispersion of data	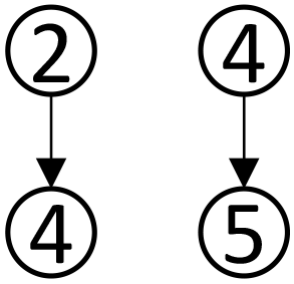
4	①Awareness of data ②Data collection ③Display of data ④Data extrapolation ⑤Calculate the relevant amount of data ⑥Using data to solve problems	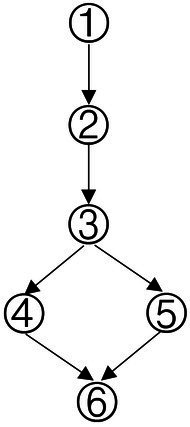	①Awareness of data ②Data collection and arrangement ③Display of data ⑥Reasoning and analysis of data	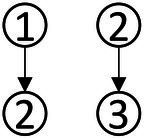
5	①Data awareness ②Collect data ③Collate the data ④Understand the data ⑤Display of data ⑥Data inference ⑦Interpret the data results ⑧Explain the data conclusion ⑨Awareness of randomness	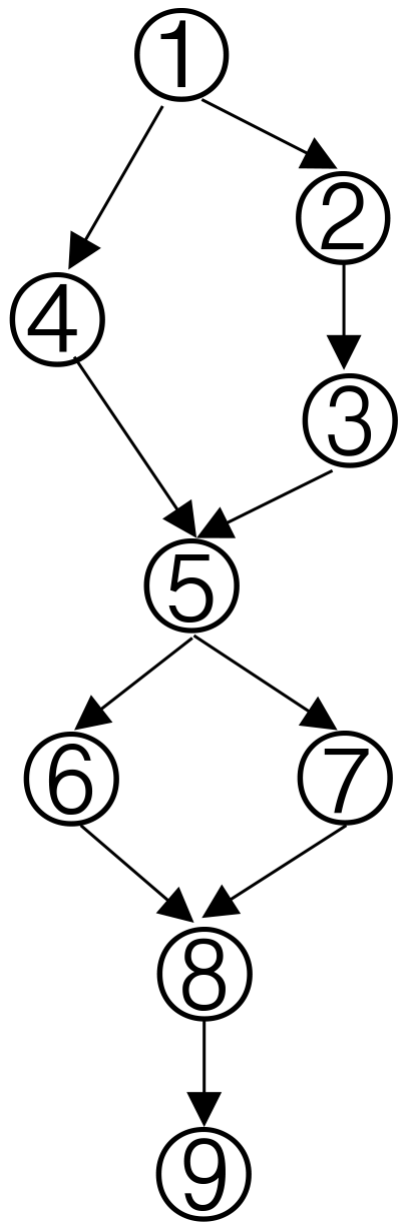	①Awareness of data ②Data collection and arrangement ③Display of data ⑥Reasoning and interpretation of data	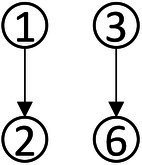

**Figure 1:**
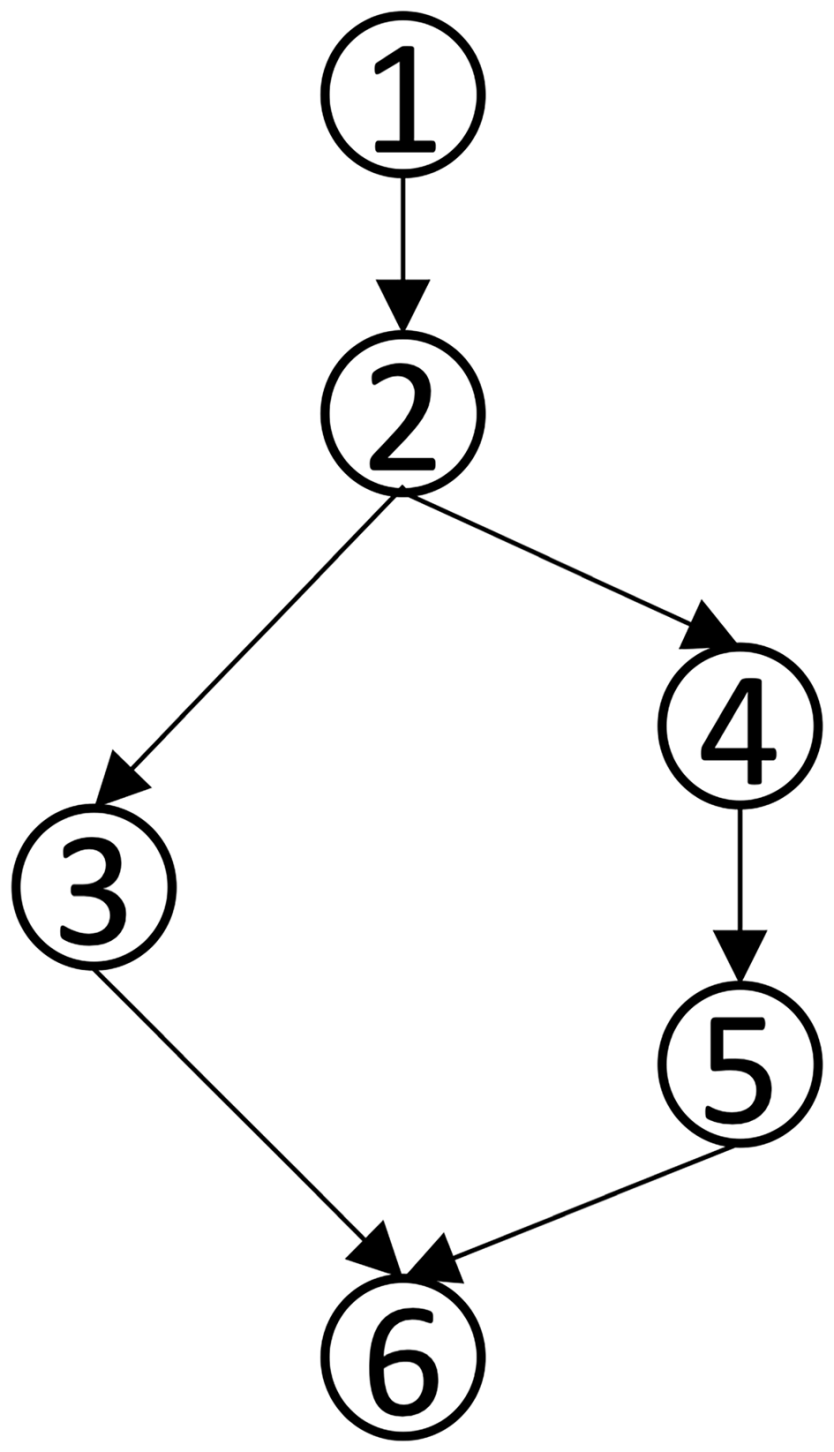
Cognitive model of data analysis ability.

Based on the common elements in Table 1 and the existing definitions of data analysis ability, the attributes of data analysis ability were extracted, which were: (1) Data awareness; (2) Data collection and sorting; (3) Data representation; (4) Data concentration; (5) Dispersion of Data; (6) Data interpretation and reasoning. According to the relationship between the extracted elements in Table 1, the structure model of data analysis ability was obtained in Figure 1.

## Cognitive diagnosis assessment of data analysis ability

### Subjects

The subjects of this study were 503 Grade 9 students from 10 classes in two middle schools in Guizhou Province, China. Guizhou is a relatively underdeveloped province in terms of education. Based on the annual proportion of admission to the secondary school entrance examination, the two schools selected belong to the upper middle level in Guizhou Province. The test was 1 h long and all Grade 9 students in both schools took the test. Informed consent was obtained from all the students and teachers before the implementation of test.

### Assessment tool

#### Construction of the Q-matrix

According to the method of constructing the Q-matrix in the Rule Space Model (RSM), the adjacency matrix was obtained according to the cognitive model shown in Figure 1. The preliminary Q-matrix can be further obtained through Boolean algebra (Tatsuoka, 2009), which was presented in Table 2.

**Table 2:** Preliminary Q-matrix of data analysis ability.

	①	②	③	④	⑤	⑥
T1	1	0	0	0	0	0
T2	1	1	0	0	0	0
T3	1	1	1	0	0	0
T4	1	1	0	1	0	0
T5	1	1	0	1	1	0
T6	1	1	1	1	1	1
T7	1	1	1	1	0	0
T8	1	1	1	1	1	0

According to Table 2, the test should be composed of 8 items. However, considering the requirements for the number of tasks completed by the students in the test, the preliminary Q-matrix in Table 2 was expanded, and 2 to 3 items were designed for the same examination mode each. The final Q-matrix including 22 items was formed, as shown in Table 3.

**Table 3:** Final Q-matrix of data analysis ability.

	A1	A2	A3	A4	A5	A6
Item1	1	0	0	0	0	0
Item2	1	1	0	0	0	0
Item3	1	1	0	1	0	0
Item4	1	1	0	1	0	0
Item5	1	1	0	1	1	0
Item6	1	1	1	1	0	0
Item7	1	1	1	1	0	0
Item8	1	1	1	1	0	0
Item9	1	1	1	1	1	0
Item10	1	1	1	0	0	0
Item11	1	0	0	0	0	0
Item12	1	1	0	0	0	0
Item13	1	1	0	0	0	0
Item14	1	1	0	1	1	0
Item15	1	1	0	1	0	0
Item16	1	1	1	0	0	0
Item17	1	1	1	1	1	0
Item18	1	1	1	0	0	0
Item19	1	1	0	1	1	0
Item20	1	1	1	1	1	0
Item21	1	1	1	1	1	1
Item22	1	1	1	1	1	1

#### Formation of assessment tools

CDA can not only reflect the internal relationship between the test items and cognitive attributes, but also demonstrate the relationship between the subject’s knowledge state and attributes (de la Torre and Chiu, 2016). One of the outstanding characteristics of CDA is its assessment structure. Through a more operational and internally consistent Q-matrix, subjects’ unobservable cognitive state can be linked to the observable item responses, which goes beyond the simple two-way list format (Wu et al., 2020). In this study, based on the final Q-matrix in Table 3, we selected 60 items from one of the large-scaled assessment tests *Trends in International Mathematics and Science Study* (TIMSS) as the first round of items selected. Through coding the attributes of these items by the five experts, 22 items with high label consistency were finally selected as the assessment tools.

#### Selection of CDM

Comparison and selection of models play a vital role in CDA process. A large number of cognitive diagnostic practices have shown that choosing an appropriate CDM is an important prerequisite for accurate diagnosis and classification of subjects (Templin and Henson, 2010). Since the theory of CDA was put forward, hundreds of measurement models varying in assumptions, parameters, mathematical principles, and actual conditions have been developed. In order to obtain a more suitable model, using the G-DINA package in the R language, this study evaluated the parameters of common models, such as Deterministic Input; Noisy ‘And’ Gate (DINA; Haertel, 1989; Junker and Sijtsma, 2001; de La Torre, 2009), Deterministic Input; Noisy ‘or’ Gate (DINO; Templin and Henson, 2006, 2010), Reduced Reparametrized Unified Model (RRUM; Hartz, 2002), Additive Cognitive Diagnosis Model (ACDM; de La Torre, 2011), Generalized Diagnostic Model (GDM; von Davier, 2014), Log-linear Cognitive Diagnosis Model (LCDM; Henson et al., 2009), Linear Logistic Model (LLM; Maris, 1999), Generalized DINA (G-DINA; de La Torre, 2011), and Mixture Model (von Davier, 2010). Based on the Q-matrix in Table 3 and test data, the relevant parameters of the CDMs were estimated, as shown in Table 4.

**Table 4:** Statistical comparison of parameters in different models.

Model	Number of parameters	Deviation	AIC	BIC
DINA	107	10735.15	10949.15	11400.75
DINO	107	10793.88	11007.88	11459.48
RRUM	162	10507.75	10831.75	11515.49
LLM	162	10503.48	10827.48	11511.22
ACDM	162	10523.89	10847.89	11531.63
GDM	121	10584.73	10826.73	11337.43
LCDM	406	10521.61	11333.61	13047.17
GDINA	447	10183.07	11077.07	12963.68
Mixed	125	10668.92	10918.92	11446.5

Two criteria are generally considered in the selection of CDMs, which are Akaike Information Criterion (AIC) and Bayesian Information Criterion (BIC). The number of parameters represents the load in the model evaluation process. The smaller the value of AIC and BIC, the smaller the load and the better the model fitting (Vrieze, 2012). Through the comparisons of AIC and BIC in Table 3, the three indicators of the GDM were the smallest, which represented the most appropriate model fit to the data.

#### GDM model

The GDM is a model that adapts to multi-level response variables and has two or more skill levels. It extends the commonly used IRT model to a multivariate, multiskilled classification model (von Davier and Yamamoto, 2004). Like other CDMs, Q-matrix is an effective part of the model. Its general form can be applied to non-integer, multi-dimensional and multi-attribute skills. It provides a more general way to specify the skill mode and the Q-matrix interact. The model (von Davier, 2008) is presented as:


$$\begin{array}{l} P_{ig}\left(x|a\right)=P\left(x|\beta_{ig},a,q_{i},\gamma_{ig}\right)\\ \;\;=\frac{\exp\left[\beta_{xig}+\sum_{k=1}^{K}x\gamma_{ikg}q_{ik}a_{k}\right]}{1+\sum_{y=1}^{m_{i}}\exp\left[\beta_{yig+}\sum_{k=1}^{K}y\gamma_{ikg}q_{ik}a_{k}\right]} \end{array}$$

where $P\left(x|\beta_{ij},a,q_{i},\gamma_{1g}\right)$ indicates that the distribution of observation variable *x* is in a given condition ($a_{1},\cdots a_{K})$, difficulty parameter $\beta_{ig}$, guessing parameter $\gamma_{ig}$ and Q-matrix $q_{i}$. $q_{ik}a_{k}=b_{k}\left(q_{i},a\right)$, however, $b\left(q_{i},a\right)=\left(q_{i1}a_{1},\cdots ,q_{iK}a_{K}\right)$ in IRT, therefore, the k*_th_* element of b $b_{k}\left(q_{i},a\right)=q_{ik}a_{k}$


$$b_{k}\left(q_{i},a\right)=\{\begin{array}{c} a_{k},\;\;\mathrm{if}\;q_{ik}=1,\\ \;\;\;0,\;\;\mathrm{if}\;q_{ik}=0. \end{array}$$

where$\;q_{ik,i=1,\cdots ,I,\;\;k=1,\cdots ,K}$ is a I×K matrix with true value $q_{i}$. This matrix associates I observed variables with K unobserved (skills) variables to determine these variables in a specific model in cognitive diagnosis. GDM is a general diagnostic model suitable for both dichotomous and multichoice data, which can model multi-dimensional mixed binary and sequential skill variables.

### Quality of assessment tool

#### Reliability of the test

Reliability represents the stability and reliability of measurement, which is one of the most important indicators of tool evaluation in the test. As a new generation of assessment, CDA has its own uniqueness that its reliability mainly focuses on the consistency index of attribute retest (Templin and Bradshaw, 2013). Similar to the Subkoviak method of decision consistency in the CTT standard reference test, this indicator is calculated by correlating the probabilities of the attribute’s mastery of the same subjects in two successive measurements, with the assumption that the probabilities of the attributes mastered by the subjects remain the same (Templin and Bradshaw, 2013). The reliabilities for different models were shown in Table 5.

**Table 5:** Reliability of data analysis ability.

Templin reliability index						
A1	A2	A3	A4	A5	A6	Mean
1	0.9275	0.7869	0.9285	0.8542	0.8658	0.8938

According to the statistics in Table 5, the test–retest reliability was estimated by repeatedly extracting from examinee’s posterior distribution to simulate repeated testing (Templin and Bradshaw, 2013). The test–retest consistency index was acceptable, with the average value reaching 0.8938. The reliability of each attribute was above 0.78, and most of them were above 0.85, indicating that the GDM model was reliable to use for the current dataset.

#### Item fit

Item fit is also the focal point in the CDM analysis. Studies have shown that whether the test data of a CDM fits the items or not directly determines the accuracy of the model’s diagnostic effect (Song et al., 2016). The conventional method to examine the fitting effect of items is the chi-square test, however, since the characteristics of the CDA do not conform to the hypothesis of the chi-square test, and the preconditions of the chi-square test do not meet, the traditional chi-square test are not appropriate to evaluate the fitting effect of items in CDM (Batanero and Díaz, 2010). In CDA, RMSEA is used to measure the fitting effect of test items by mainly comparing the square root error of observed response and predicted response under the different potential classifications. The calculation formula of RMSEA for item *j* is:


$$RMSEA_{j}=\sqrt{\underset{k}{\sum }\underset{c}{\sum }\pi \left(\theta_{c}\right){\left(P_{j}\left(\theta_{c}\right)-\frac{n_{jkc}}{N_{jc}}\right)}^{2}}$$

where $\pi \left(\theta_{c}\right)\;$represents the classification probability of the potential trait level of type c, and $P_{j}\;$represents the probability estimated by the item response function. $n_{jkc}\;$represents the expected number of people at the k*_th_* dimension of the c*_th_* potential trait level in the j*_th_* item, and $N_{jc}\;$represents the expected number of people at the c*_th_* potential trait level. Through the calculation of residual information, the residual information of the test items was shown in Table 6.

**Table 6:** RMSEA information of the test items.

Item	Item 1	Item 2	Item 3	Item 4	Item 5	Item 6	Item 7	Item 8
RMSEA	0.0176	0.0406	0.1041	0.0839	0.0989	0.0807	0.1151	0.0812
Item	Item 9	Item 10	Item 11	Item 12	Item 13	Item 14	Item 15	Item 16
RMSEA	0.1028	0.0673	0.0684	0.0486	0.1107	0.0942	0.0799	0.0692
Item	Item 17	Item 18	Item 19	Item 20	Item 21	Item 22	\	\
RMSEA	0.1857	0.1103	0.4106	0.2119	0.1923	0.2511	\	\

The closer the RMSEA value is to 0, the smaller the deviation of the fit and the better the fitting effect. The critical value of RMSEA is normally set to 0.1. Values greater than 0.1 for RMSEA is an indication of poor item fit (Oliveri and von Davier, 2011). According to this standard, the GDM model was still the one with the best fit in the test items since the RMSEA values for all the test items were less than 0.1. Only items 3, 7, 9, 13, 17, 18, 19, 20, 21, 22 had the values slightly greater than 0.1. Therefore, the GDM model still had the most appropriate item level fit in this study.

## Assessment result

### Probability of mastery of attributes of data analysis ability

**Figure 2:**
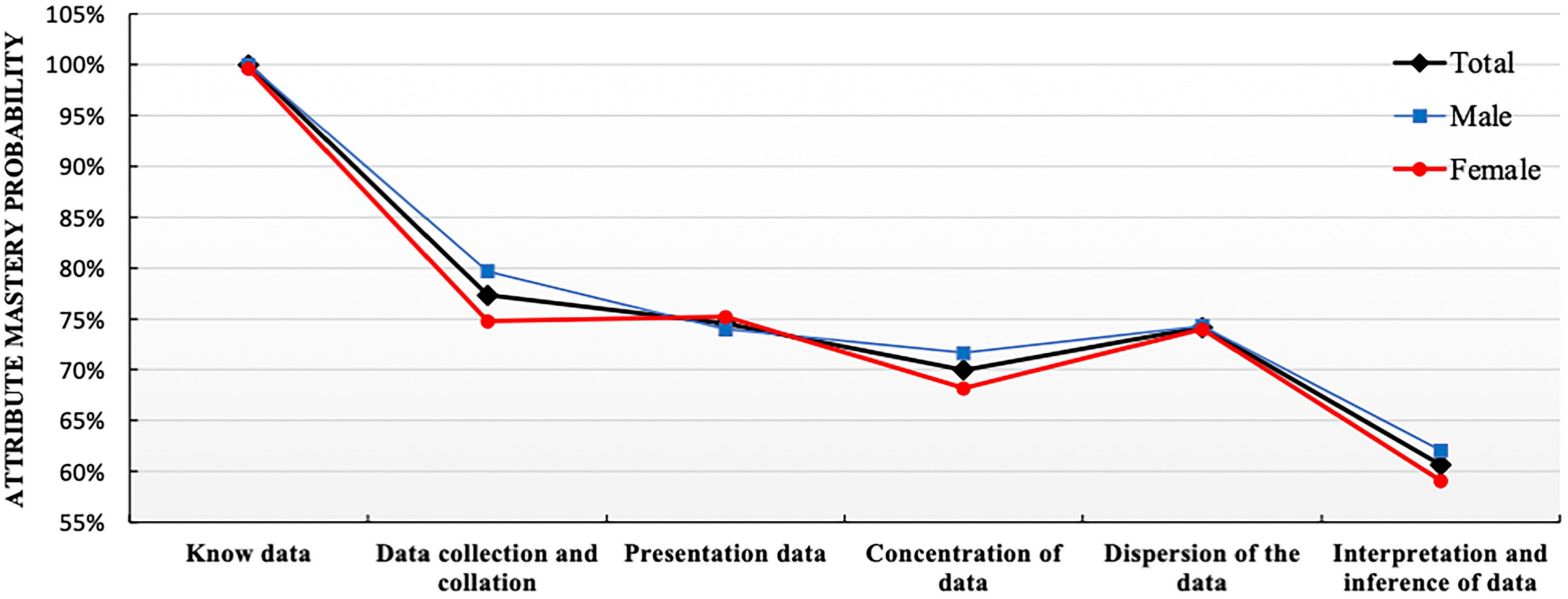
Distribution diagram for attribute mastery probability of data analysis ability.

The probability of each student’s attribute mastery can be obtained through the assessment of the model in CDA. To further analyze the gender differences in students’ mastery of attributes, comparison analyses of the attribute mastery probability were conducted on genders and the mastery probabilities of the six attributes were obtained as shown in Figure 2.

Figure 2 showed that the mastery probability of the attribute *Know data* was the highest, reaching 100%, which indicated that almost all students had the basic data awareness. The probability of mastering these four attributes, *Data collection and collation, Data presentation, Concentration of data,* and *Dispersion of the data*, were quite similar at about 70%. Among these four, the probabilities of mastering attributes of *Data collection and collation* and *Dispersion of the data* were slightly higher. Last, the mastery probability of *Interpretation and inference of data* was the lowest, about 60%.

Overall, in Figure 2, what we can observe was that there was no obvious gender difference in attribute mastery probability, especially the probability of mastering *Know data*, *Data presentation* and *Dispersion of data* were almost the same for both male and female students. In terms of *Data collection and collation*, *Concentration of data* and *Interpretation and inference of data*, the probabilities of male students mastering attributes were slightly higher than these for female ones, showing certain advantages for male students.

### Construction of learning progression for data analysis ability

**Figure 3:**
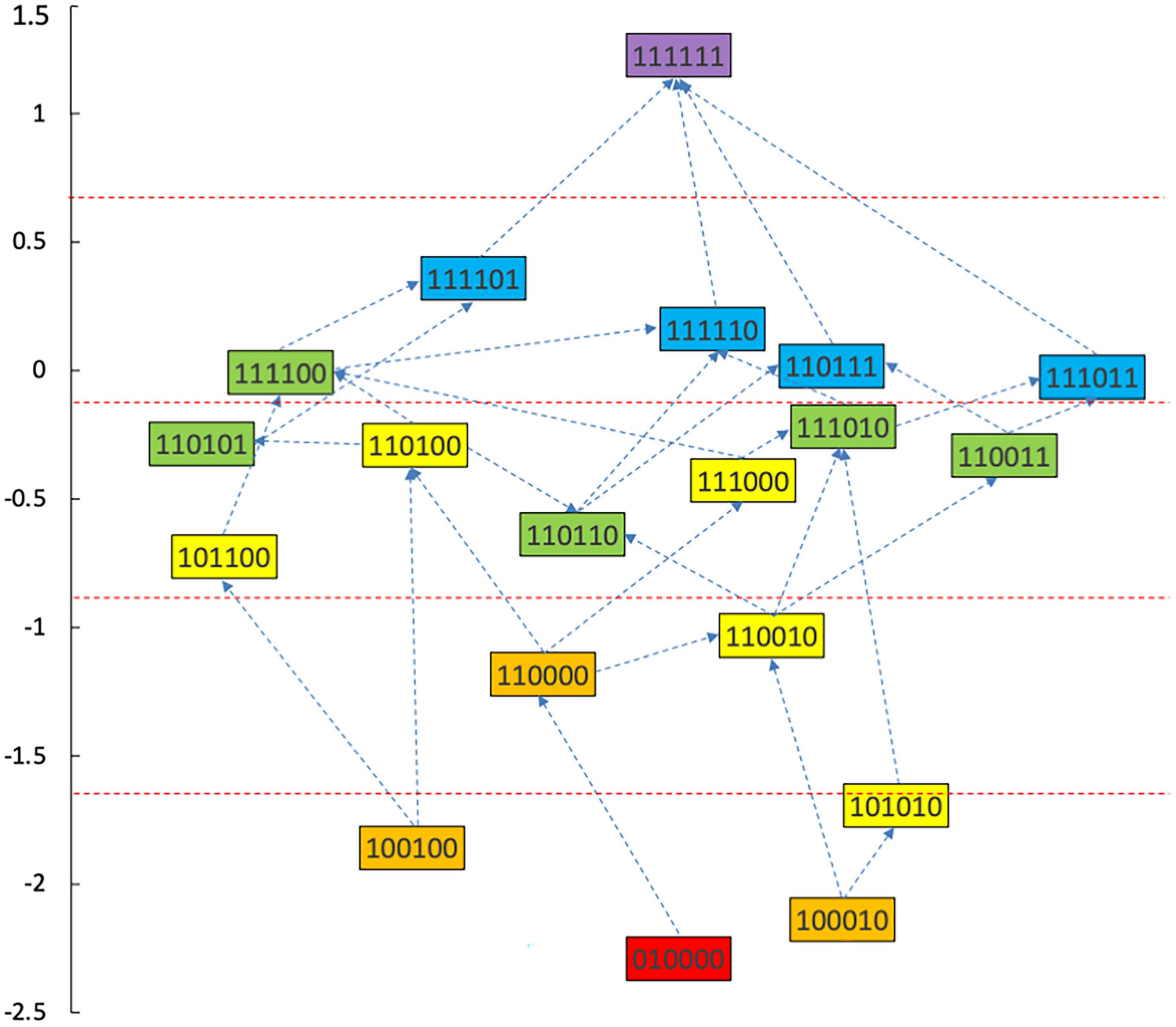
Advanced diagram of data analysis ability progression.

With a combined method of learning progression construction with Item Response Theory (IRT), students’ abilities were calculated in each type of knowledge state, and students in different knowledge state categories were divided (Wu et al., 2021). The ability value 1.37 was the highest for the knowledge state (111111), and the ability value −2.41 was the lowest for the knowledge state (010000). We have divided the interval (−2.5, 1.5) into 5 levels and the diagram for learning progression was shown in Figure 3.

According to the attributes of the knowledge states in the different stages in Figure 3, the learning progression of the ladder were defined, and the divisions of learning progression level were organized in Table 7. It provided a more reliable theoretical basis for student learning, teacher teaching, textbook compilation, and test compilation.

**Table 7:** Divisions of learning progression levels of data analysis ability.

Level	Content definition	Attribute mastery
1	Students can pay attention to the core data in problem solving, and be able to distinguish different data, recognize the role of data in problem solving, and have a certain sense of data	Preliminary mastery of attributes ①
2	Students are able to collect data according to reasonable methods, and carry out preliminary sorting and statistics on the collected data, and carry out preliminary data management.	On the basis of mastering ①, further master the attributes ②
3	Students can properly represent the data, such as using histograms, line graphs, etc., and can freely convert between different representations; can calculate the average, mode, and median of the data and experience different quantities specialty	On the basis of mastering ① and ②, further master ③ and ④ attributes
4	Students can calculate the degree of dispersion of data, such as variance, standard deviation, range, etc., based on the amount of concentration, and appreciate the meaning of the degree of dispersion of data. Infer the meaning expressed by the data and draw useful conclusions.	On the basis of mastering the attributes ① to ④, grasp the attributes ⑤ and ⑥ initially
5	On the basis of mastering the concepts and operations of integers, measurement, plane geometry, data, probability, and preliminary statistics, students have further mastered the basic concepts and operations of elementary algebra, and can solve practical problems related to equations, inequalities, and functions	Master all attributes

### Personalized analysis of data analysis ability

**Figure 4:**
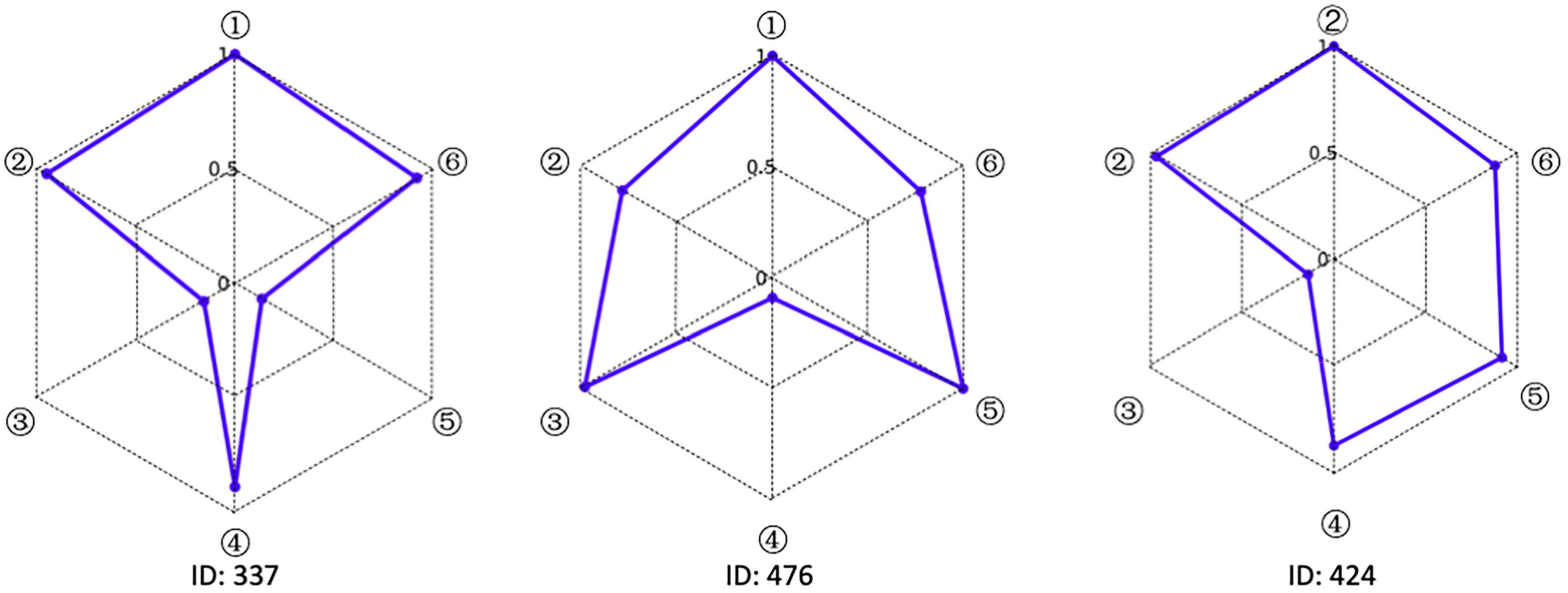
Radar chart of the mastery results of students’ data analysis ability.

Accurately depicting the knowledge state of each student is the greatest advantage of CDA. To fully illustrate the fine-grained information that CDA can provide for each student, three students numbered 337, 476 and 424 were selected for the analysis. The radar chart of their attribute mastery was shown in Figure 4.

The characteristic of the three students shown in Figure 4 was that they correctly answered the same number of questions. If through the traditional evaluation methods, these students were considered to have the same total score, showing that there was no difference among them. However, in CDA, differences were still obvious in Figure 3. Student No. 337 had mastered attributes ①, ②, ④, ⑥; student No. 476 had mastered attributes ①, ②, ③, ⑤, ⑥, but not fully mastered attributes ② or ⑥, whose probability of attribute mastery was approximately 80%; and student No. 424 had mastered the attributes ①, ②, ④, ⑤, ⑥. Therefore, what we can conclude was that these three students not only differed in the type of attribute mastery, but also in the number and degree of attribute mastery. These results offered more fine-grained information for students’ personalized learning.

## Discussion

This study constructed a reasonable cognitive model for students’ data analysis ability based on the expert method. Results showed that students had a good attribute mastery of *Know data*, but students’ mastery of *Interpretation and inference of data* was relatively poor. Data analysis ability can be clearly divided into five levels of learning progression and students with the same scores differed obviously in the knowledge state.

As one of the essential core qualities of modern citizens, the importance of data analysis ability has been recognized by all walks of life. The accurate assessment of data analysis ability is a topic that is worthy further exploration. As a new generation of assessment theory, CDA is designed to detect students’ specific knowledge structure or operational skills in a certain field, so as to provide students with more fine-grained diagnostic information on cognitive strengths and weaknesses (Leighton and Gierl, 2007). It is essentially a diagnosis of cognitive attributes, and the construction of attributes plays a vital role in the assessment process (Wu et al., 2020). However, most of the extant research have only considered the division of cognitive attributes without taking their prerequisite relationship into account. Starting from expert method, this study constructed a cognitive model while having the prerequisite relationship between attributes into account. The model that was formed has provided a theoretical basis for clarifying the relationship between the attributes of data analysis ability and further guiding the teaching and students’ personal assessment. It provided a more standardized research method for the assessment of students’ data analysis abilities, which was more in line with the research paradigm of CDA.

In this study, we also explored the learning path and learning progression of students’ data analysis ability. The learning path depicted student’s thinking and learning in a specific field of mathematics, reflecting student’s learning process through a series of instructive tasks. These tasks were designed to promote the development of students’ mental process and thinking level (Clements and Sarama, 2012). The learning path reflects the trend of students’ actual learning progress, rather than focusing on subject knowledge, which distinguishes the learner’s logic from the subject logic and plays an intermediary role in the selection of learning goals and methods (Corcoran et al., 2009). The realization of different learning objectives needs to be supported by the corresponding learning paths, and different learning paths will determine the choice of learning methods. In the selection of learning methods, learning path distinguishes the student’s “voice” from the subject’s perspective, emphasizes the development of students’ cognitive order, and further clarifies the importance of learners in guiding future teaching, curriculum and assessment (Confrey, 2006). Students are all in different levels of learning paths, with various learning resources and learning environment around, therefore, a selection of a suitable learning path and learning method according to their individual conditions and backgrounds will be more appropriate. Learning path helps learners choose appropriate teaching activities, tasks, tools, interaction and evaluation methods, and promotes students to gradually master increasingly complex concepts (Confrey et al., 2009). It combines the evaluation results of each student with the corresponding learning mode, extracts the formative evaluation results from the summative evaluation data, and provides a basis for students to formulate their personalized learning plans.

This study provided a more complete and standardized research method, constructed a cognitive model of data analysis ability, and made contributions to the theory as well as methods to a certain degree. However, due to the limited material and financial resources, this study also had some shortcomings inevitably. First, the sample size available was limited. With only approximately 500 students in China, the result of the study lacked generalizability to some extent. In addition, any assessment cannot make without the discipline itself. The evaluation content should be analyzed based on the characteristics of the discipline (Zhang et al., 2019). Last, a longitudinal study with similar method and approach is recommended in the future to verify the result reliability and validity (Zhan et al., 2019; Wu et al., 2020).

## Data availability statement

The data analyzed in this study is subject to the following licenses/restrictions: The datasets generated during and/or analyzed during the current study are available from the corresponding author on reasonable request. Requests to access these datasets should be directed to XW, 18198689070@126.com.

## Ethics statement

Ethical review and approval was not required for the study on human participants in accordance with the local legislation and institutional requirements. The patients/participants provided their written informed consent to participate in this study.

## Author contributions

XW designed the study and wrote this manuscript. YZ contributed to the manuscript writing and the continued revision provided by the reviewers. XT collected the data and wrote this manuscript. RW and TX have revised the language of the article and provided comments. All authors contributed to the article and approved the submitted version.

## Funding

This work was support by 2021 Humanities and Social Science Research project of Ministry of Education of China: Cognitive Diagnosis and Evaluation of Mathematics Core Literacy (21YJC880102). Teacher Education “JIEBANGLINGTI” Project of Northeast Normal University: Learning Progression Construction and Learning Path Analysis Based on Cognitive Diagnosis (JSJY20220305).

## Conflict of interest

The authors declare that the research was conducted in the absence of any commercial or financial relationships that could be construed as a potential conflict of interest.

## Publisher’s note

All claims expressed in this article are solely those of the authors and do not necessarily represent those of their affiliated organizations, or those of the publisher, the editors and the reviewers. Any product that may be evaluated in this article, or claim that may be made by its manufacturer, is not guaranteed or endorsed by the publisher.
